# The association between TNF-receptors (TNFR1 and TNFR2) and mortality as well as kidney function decline in patients with chronic kidney disease

**DOI:** 10.48101/ujms.v129.10726

**Published:** 2024-11-25

**Authors:** Per Wändell, Tobias Feldreich, Anders Larsson, Philip A. Kalra, Johan Ärnlöv, Toralph Ruge, Axel C. Carlsson

**Affiliations:** a Department of Neurobiology, Care Sciences and Society, Karolinska Institutet, Huddinge, Sweden; b School of Health and Welfare, Dalarna University, Falun, Sweden; c Department of Medical Sciences, Uppsala University, Uppsala, Sweden; d Department of Renal medicine, Salford Royal, Northern Care Alliance NHS Foundation Trust, Salford, UK; e Department of Emergency and Internal Medicine, Skånes University Hospital, Malmö, Sweden; f Department of Clinical Sciences Malmö, Lund University & Department of Internal Medicine, Skåne University Hospital, Malmö, Sweden; g Academic Primary Health Care Centre, Stockholm Region, Stockholm, Sweden

**Keywords:** Chronic kidney disease, Soluble Tumor Necrosis Factor Receptor (TNFR), mortality, decline in kidney function, cardiovascular diseases, cardiovascular risk factors

## Abstract

**Background:**

Higher circulating levels of tumor necrosis factor (TNF) alpha receptors 1 (TNFR1) and 2 (TNFR2) are associated with increased long-term mortality and impaired kidney function.

**Aim:**

To study associations between levels of TNFR1 and TNFR2 and all-cause mortality as well as estimated glomerular filtration rate (eGFR) decline.

**Population and methods:**

Patients with chronic kidney disease (CKD) stages 3–5 in the Salford Kidney Study were included. Associations between one standard deviation increase in **plasma** TNFR1 and TNFR2 and mortality were estimated by Cox regression models with hazard ratios (HRs) and 95% confidence intervals adjusted for age, sex, eGFR based on creatinine and cystatin C, urine-protein, C-reactive protin, cardiovascular comorbidity, smoking habits, and diabetes. Differences in eGFR decline in relation to **plasma** TNFR1 and TNFR2 were estimated by both linear and logistic regression models, with regression coefficients and odds ratios (ORs).

**Results:**

Univariate models showed significant associations between TNFR1 **(*n* = 985)** and TNFR2 **(*n* = 988)** and all-cause mortality based on 7424 person-years at risk, but in the fully adjusted models with continuous variables significant only for TNFR2 HR 1.17 (1.03–1.34), but with a borderline value for TNFR1 HR 1.15 (1.00–1.31). For rapid decliners, that is, eGFR decline in highest TNFR-receptor quartile versus quartiles 1–3, the decline was 1.60% per month (interval 0.78–10.99). For eGFR decline in continuous models, the fully adjusted ORs were for TNFR1 1.29 (0.92–1.81) and for TNFR2 1.33 (0.90–1.98).

**Conclusions:**

TNFR2 was associated with mortality, but TNFR1 was not, although showing a borderline value. Neither TNFR1 nor TNFR2 predicted decline in kidney function. TNFR1 and TNFR2 portray interesting aspects in patients with CKD, but the clinical utility seems limited.

## Introduction

The investigation of biomarkers has become integral to the comprehensive understanding of disease trajectories and prognostic assessments, particularly in the intricate context of chronic kidney disease (CKD). Within this milieu, tumor necrosis factor receptors 1 and 2 (TNFR1 and TNFR2) have emerged as potential indicators with implications for mortality (1–3) and the progressive decline of kidney function (3–5). As signaling mediators in the tumor necrosis factor (TNF) pathway, these receptors are implicated in inflammatory processes (6), which are pivotal in CKD progression (7).

Tumor Necrosis Factor alpha (TNF alpha) is produced by macrophages/monocytes during acute inflammation and is involved in many signaling events within cells through TNFR, mainly leading to necrosis or apoptosis (8). Membrane TNFRs are expressed on the surface of cells as transmembrane proteins, and the cleaving of TNFR leads to liberated and soluble TNFR of two types, TNFR1 and TNFR2 (6). TNFR1 is expressed on most cells, and the signaling pathway by TNF via TNFR1 mainly triggers pro-inflammatory pathways (9). In contrast, TNFR2 only signals not only for antiapoptotic reactions but also to induce TNF receptor adaption factor 2 (TRAF2) degradation, where TRAF2 is a key mediator in signal transduction of both TNFR1 and TNFR2 (10). TRAFs are a family of structurally related proteins that transduce signals from members of TNFR superfamily and various other immune receptors (11). Higher circulating TNFR1 has been shown to independently predict progression to a worse glomerular filtration rate (GFR) category, to CKD incidence in elderly individuals (4), and also to be associated with a higher all-cause mortality risk among individuals with both high and low levels of systemic inflammation (2). The all-cause mortality risk includes both cancer and cardiovascular mortality and is more common in elderly individuals. TNFR1 and TNFR2 are also associated with worse kidney function and mortality in patients with diabetes (1, 3, 12). In short, the association between TNFR1 and TNFR2 has mainly been conducted in elderly community dwelling individuals, and studies on populations with patients with CKD are limited; it would be of interest to assess the value of TNFR1 and TNFR2 in this patient group.

The aim of this study was to scrutinize the utility of TNFR1 and TNFR2 as discerning markers, seeking to delineate their associations with mortality and the trajectory of renal function decline in patients in a large CKD cohort.

## Methods

### Study population

The Salford Kidney Study is an ongoing prospective study that started in 2002, and at the time of this study, it only included patients with CKD stages 3–5 but not on dialysis therapy, with the intention to investigate the development and outcomes of kidney disease and associated comorbidity (13). Subsequent amendments have enabled recruitment of patients receiving renal replacement therapy (RRT).

Briefly, a random sample of all patients with estimated glomerular filtration rate (eGFR) >10 and <60 mL/min/1.73 m2 referred for renal care to Salford Royal Hospital renal services were approached to take part in the study. Patients with previous evidence of RRT, that is, dialysis or kidney transplant, were excluded from this study. In total, 985 individuals with measured TNFR1 and 988 individuals with measured TNFR2 were included.

### Clinical data

At enrollment into the study and at annual follow-up, demographic, clinical, and laboratory data were obtained from the electronic patient records, a continually updated electronic healthcare system, as well as patient interview and detailed clinical examination. Age, sex, and blood pressure were recorded, and self-reported smoking status was classified as current smokers, ex-smokers, or non-smokers. Ex-smokers who had stopped smoking during the last year before enrollment were categorized as current smokers. Comorbidity variables available for analysis included previous cardiovascular diseases, diabetes, and heart failure. Blood pressure was recorded by an automated oscillometer (CARESCAPE Monitor B850 or B650, General Electric Healthcare), consciousness was determined according to the Reaction Level Scale (14). Cause of death data were obtained from the Office of National Statistics.

### Laboratory measurements

Bio-samples were collected at enrollment into the study and stored at −80 C. Serum creatinine measurements were performed using a blank rated and compensated Jaffé reaction with a Roche Modular analyzer (Roche Diagnostics, Rotkreuz, Switzerland), which was isotope-dilution mass spectrometry-calibrated, at routine annual clinic visits. In addition to internal quality control, the laboratory participates in the UK National External Quality Assessment Scheme. eGFR-creatinine was estimated using the Chronic Kidney Disease Epidemiology Collaboration creatinine equation (without ethnic factor) from 2009 (15). Cystatin C was measured on a BS380 instrument (Mindray, Shenzhen, China), with cystatin C reagents from Gentian (Moss, Norway). eGFR based on cystatin C was also calculated using the Caucasian, Asian, pediatric, and adult (CAPA) cohorts equation (16). Urine protein concentration was collected for 24 h.

TNF receptor 1 and TNF receptor 2 were analyzed with commercial sandwich enzyme-linked immunosorbent assay (ELISAs) (DY225 and DY276, R&D Systems, Minneapolis, MN, USA) according to the recommendations of the manufacturer. The total coefficients of variation for the ELISAs were approximately 6%.

### Follow-up outcomes

All-cause mortality was used as outcome. eGFR difference was calculated as the difference between eGFR-creatinine at baseline and at the last visit before exiting the study or last follow-up and expressed as % decrease per month, thus an eGFR difference >0 indicated an eGFR decline (17).

### Statistical analysis

Variables with skewed distribution, that is, TNFR, U-protein, C-reactive protein (CRP), and eGFR cystatin, according to the Shapiro-Wilks test (*W* < 0.95), were log-transformed to promote normal distribution. Missing data were imputed using the imputation via chained equations (ICE) command in STATA (missing data were 17 for age, gender, diabetes, mean systolic blood pressure, mean diastolic blood pressure, smoking, heart failure, and previous cardiovascular event; 186 for U-protein; 134 for C-reactive protein; 24 for eGFR-Cystatin C; and 31 for eGFR-creatinine). Associations at baseline were evaluated in linear regression models or Analysis of variance (ANOVA). Spearman’s correlation test was performed.

The associations of baseline TNFR1 and TNFR2 and total mortality were analyzed with Cox proportional hazard regression in univariable and in five multivariable models (A–E): Model A: adjusted for age and gender as both mortality and kidney function vary with age and sex.

Model B adjusted for age, gender, and C-reactive protein as inflammation has effects on both kidney function and the risk of death.

Model C: adjusted for age, gender, systolic blood pressure, diastolic blood pressure, smoking status, heart failure, diabetes, and prevalent cardiovascular disease as established risk factors for cardiovascular disease are risk factors for the kidney and for death.

Model D. Model A + B + C combined.

Model E. Model A + B + C + baseline eGFR and urine protein concentration as an ideal risk factor in CKD should add information in addition to clinically used kidney markers.

Associations between baseline TNFR1 and TNFR2, and eGFR differences over time were also analyzed in linear regression models A-E, as shown earlier. Associations between TNFR1 and TNFR2 at baseline, and eGFR difference, and risk of being a rapid eGFR-decliner (defined as quartile 4 versus quartile 1–3 of eGFR difference over follow-up) were analyzed in logistic regression in models A-E as shown earlier, with odds ratios (ORs). We also performed Nelson-Aalen plots of cumulative incidence of mortality by participants divided into quartiles according to concentrations of TNFR1 and TNFR2.

A *p*-value of <0.05 was considered statistically significant. The dataset was handled, and calculations were performed with Stata 16 (Stata Corp., College Station, TX, USA).

## Results

Baseline characteristics of the included individuals with values of TNFR1 and TNFR2 are shown in Tables 1 and 2, also including data on the quartiles of TNFR1 and TNFR2. Higher quartiles of TNFR1 and TNFR2 were associated with higher values of creatinine, cystatin C, and U-protein, and lower values of eGFR for both creatinine and cystatin C. The Spearman correlation between TNFR1 and TNFR2 was 0.75, *P* < 0.001, showing that they are highly correlated but independent variables.

**Table 1:** Baseline characteristics of the study population (n = 987) and in quartiles of TNFR1 (pg/mL).

Variable	Quartiles					*P*
	All	1 (lowest)	2	3	4 (highest)	
Number of patients	*n* = 987	*n* = 248	*n* = 246	*n* = 247	*n* = 246	
	**Mean (SD)**					
TNFR1 (pg/mL)	4560 (2572)	2047 (495)	3362 (356)	4797 (493)	8040 (2431)	-
Age (years)	63 (14)	62 (13)	62 (14)	64 (15)	65 (15)	0.11
Creatinine (µmol/L)	228 (127)	140 (41)	192 (67)	245 (112)	336 (157)	<0.001
eGFR creatinine (mL/min/1.73 m^2^)	31 (16)	46 (16)	33 (12)	26 (13)	19 (11)	<0.001
Cystatin C (mg/L)	2.7 (1.2)	1.9 (0.9)	2.4 (0.9)	2.9 (0.9)	3.6 (1.1)	<0.001
eGFR Cystatin C (mL/min/1.73 m^2^)	26 (15)	40 (17)	27 (11)	22 (11)	16 (9.2)	<0.001
Systolic blood pressure (mmHg)	138 (21)	137 (22)	136 (20)	137 (22)	140 (22)	0.16
Diastolic blood pressure (mmHg)	76 (12)	77 (12)	75 (12)	75 (12)	75 (12)	0.09
U-protein (g/L)	0.48 (0.83)	0.20 (0.36)	0.41 (0.74)	0.54 (0.88)	0.75 (1.06)	<0.001
C-reactive protein (mg/L)	7.97 (15.88)	4.71 (6.85)	6.22 (9.62)	7.33 (12.34)	13.61 (25.95)	<0.001
	***N* (%)**					***P***
Females	363 (37)	90 (37)	78 (32)	107 (44)	88 (37)	0.07
Previous cardiovascular disease	283 (29)	62 (26)	78 (32)	69 (28)	74 (31)	0.45
Smoking, current or previous	627 (65)	146 (60)	155 (64)	164 (67)	162 (67)	0.35
Diabetes	286 (29)	50 (21)	80 (33)	79 (32)	77 (32)	0.007
Heart failure	154 (16)	20 (8)	39 (16)	41 (17)	54 (22)	0.008

SD: standard deviation; eGFR: estimated glomerular filtration rate.

The *P*-value shows differences between quartiles of TNFR1.

**Table 2:** Baseline characteristics of the study population (n = 984) and in quartiles of TNFR2 (pg/mL).

Variable	Quartiles					P
	All	1 (lowest)	2	3	4 (highest)	
Number of patients	*n* = 984	*n* = 247	*n* = 245	*n* = 246	*n* = 246	
	**Mean (SD)**					
TNFR2 (pg/mL)	12441 (7062)	6583 (1274)	9725 (903)	13029 (1092)	20453 (9480)	-	
Age (years)	63 (14)	62 (14)	64 (14)	63 (14)	65 (14)	0.015
Creatinine (µmol/L)	229 (127)	143 (49)	191 (74)	252 (114)	327 (157)	<0.001
eGFR creatinine (mL/min/1.73 m^2^)	31 (16)	46 (16)	33 (13)	25 (12)	19 (10)	<0.001
Cystatin C (mg/L)	2.7 (1.2)	1.9 (1.0)	2.5 (0.9)	3.0 (1.0)	3.4 (1.0)	<0.001
eGFR Cystatin C (mL/min/1.73 m^2^)	26 (15)	39 (17)	28 (13)	20 (10)	17 (9.2)	<0.001
Systolic blood pressure (mmHg)	138 (21)	136 (21)	135 (20)	139 (21)	140 (23)	0.39
Diastolic blood pressure (mmHg)	76 (12)	77 (12)	74 (11)	76 (12)	75 (12)	0.45
U-protein (g/L)	0.48 (0.83)	0.19 (0.31)	0.36 (0.59)	0.51 (0.69)	0.86 (1.28)	<0.001
C-reactive protein	7.99 (15.90)	4.27 (6.89)	6.15 (9.78)	8.40 (15.76)	13.25 (24.13)	<0.001
	***N* (%)**					***P***
Females	363 (37)	87 (36)	91 (38)	103 (42)	82 (34)	0.24
Previous cardiovascular disease	283 (29)	60 (25)	64 (27)	76 (31)	83 (34)	0.16
Smoking, current or previous	625 (65)	143 (59)	150 (63)	162 (66)	170 (70)	0.06
Diabetes	286 (29)	59 (24)	67 (28)	86 (35)	74 (30)	0.06
Heart failure	154 (16)	33 (14)	30 (13)	32 (13)	59 (24)	0.001

SD: standard deviation; eGFR: estimated glomerular filtration rate.

The *P*-value shows differences between quartiles of TNFR2.

Mean follow-up time was 7.5 years (SD ± 4 years), and 555 (56%) patients died. In the initial unadjusted model, we observed a significant increasing hazard ratio in all quartiles. The association between TNFR1 and TNFR2 values and mortality after 7432 and 7405 person-years at risk, respectively, is shown in Table 3. The increase of TNFR1 and TNFR2 values by one standard deviation was associated with unadjusted HRs of 1.42 and 1.40, respectively, and in fully adjusted models of 1.15 (not significant) and 1.17, respectively. Divided into quartiles, TNFR2 quartiles 2 and 4 versus quartile 1 were statistically significant in the fully adjusted model, but TNFR1 was non-significant in fully adjusted models for quartiles 2, 3, and 4 versus quartile 1. Nelson-Aalen plots of cumulative incidence of mortality by participants divided into quartiles according to concentrations of TNFR1 and TNFR2, respectively, are shown in Supplementary Figures 1 and 2, with distinctive differences for the curves of quartiles for TNFR2, and for TNFR1 for quartiles 1 and 4, while curves for quartiles 2 and 3 were rather similar.

**Table 3:** Associations between TNFR1 and TNFR2 and all-cause mortality risk in Cox regression models.

	Continuous	Quartiles			
	1 SD-increase HR (95% CI)	1 (lowest) HR (95% CI)	2 HR (95% CI)	3 HR (95% CI)	4 (highest) HR (95% CI)
**TNFR1**					
Number of events/numbers at risk	542/971	100/242	134/243	144/245	164/241
Unadjusted model	1.42*** (1.31–1.55)	referent	1.52** (1.18–1.96)	1.78*** (1.38–2.28)	1.51*** (1.96–3.20)
Model A (age, gender)	1.46*** (1.34–1.60)	referent	1.55** (1.20–2.00)	1.79*** (1.39–2.31)	2.61*** (2.02–3.36)
Model B (inflammation)	1.40*** (1.28–1.54)	referent	1.51** (1.17–1.95)	1.73*** (1.34–2.23)	2.37*** (1.83–3.08)
Model C (CVD risk factors)	1.43*** (1.30–1.57)	referent	1.49** (1.15–1.93)	1.73*** (1.34–2.24)	2.42*** (1.87–3.14)
Model D (Model A+B+C)	1.38*** (1.25–1.53)	referent	1.46** (1.13–1.89)	1.68*** (1.30–2.18)	2.22*** (1.70–2.90)
Model E (all combined + eGFR + urine Protein)	1.15 (1.00–1.31)	referent	1.21 (0.91–1.59)	1.17 (0.85–1.60)	1.31 (0.92–1.87)
**TNFR2**					
Number of events/numbers at risk	542/968	91/244	127/237	149/244	175/243
Unadjusted model	1.40*** (1.30–1.51)	referent	1.62*** (1.24–2.10)	2.00*** (1.54–2.59)	3.04*** (2.36–3.90)
Model A (age, gender)	1.48*** (1.36–1.60)	referent	1.60** (1.23–2.10)	2.00*** (1.53–2.61)	3.02*** (2.34–3.89)
Model B (inflammation)	1.35*** (1.20–1.51)	referent	1.55** (1.18–2.02)	1.90*** (1.45–2.49)	2.76*** (2.12–3.58)
Model C (CVD risk factors)	1.44*** (1.32–1.57)	referent	1.63*** (1.25–2.14)	1.92*** (1.47–2.52)	2.86*** (2.21–3.69)
Model D (A + B + C)	1.40*** (1.29–1.53)	referent	1.58** (1.21–2.07)	1.81*** (1.38–2.38)	2.64*** (2.03–3.44)
Model E (all combined+ eGFR + urine-protein)	1.17* (1.03–1.34)	referent	1.33* (1.00–1.76)	1.32 (0.96–1.82)	1.64** (1.16–2.32)

HR: hazard ratio; CI: confidence interval; SD: standard deviation; eGFR: estimated glomerular filtration rate; CVD: cardiovascular disease

Hazard ratio calculated based on 7424 person-years at risk;

*** *P* < 0.001;

** *P* < 0.01;

* *P* < 0.05.

Model A: Adjusted for age and gender. Model B: Adjusted for age, gender, and C-reactive protein. Model C: Adjusted for age, gender, systolic blood pressure, diastolic blood pressure, smoking status, heart failure, diabetes, and prevalent cardiovascular disease.

Model D: Model A + B + C combined. Model E: Model A+B+C+baseline eGFR and u-protein.

Values of eGFR differences over time (mean time 4.37 years) are shown in Table 4, also by quartiles, with quartiles 3 and 4 showing decline in eGFR and quartile 1 showing increase in eGFR. For continuous models and quartile 4 versus quartiles 1–3 (Table 5), the regression coefficients were significantly associated with eGFR except in the fully adjusted model (with addition of baseline eGFR and U-protein) for both TNFR1 and TNFR2.

**Table 4:** Mean eGFR difference in all participants (in % per month) and in quartiles of eGFR difference for TNFR1 and TNFR2.

	*N*	Mean (% per month)	Interval (min-max)	Comment
**TNFR1**				
All	455	0.40	−7.34; 10.99	7.34% eGFR increase to 10.99% eGFR decline
Quartile 1	114	−0.72	−7.34; −0.007	7.34 to 0.007% eGFR increase
Quartile 2	114	0.18	−0.007; 0.34	0.007% eGFR increase to 0.34% eGFR decline
Quartile 3	114	0.55	0.34; 0.77	0.34 to 0.77% eGFR decline
Quartile 4 (’rapid decliner’)	113	1.60	0.78; 10.99	0.78 to 10.99% eGFR decline
Quartiles 1–3	342	0.0051	−7.34; 0.77	7.34% eGFR increase to 0.77% eGFR decline
**TNFR2**				
All	453	0.40	−7.33; 10.99	7.33% eGFR increase to 10.99% eGFR decline
Quartile 1	114	-0.72	−7.33; −0.007	7.33 to 0.007% eGFR increase
Quartile 2	113	0.18	−0.004; 0.34	0.004% eGFR increase to 0.34% eGFR decline
Quartile 3	113	0.55	0.34; 0.77	0.34 to 0.77% eGFR decline
Quartile 4 (’rapid decliner’)	113	1.60	0.78; 10.99	0.78 to 10.99 % eGFR decline
Quartiles 1–3	340	0.0038	−7.33; 0.77	7.33% eGFR increase to 0.77% eGFR decline

eGFR: estimated glomerular filtration rate.

eGFR based on cystatin C was also calculated using the CAPA equation. The differences in time between the measurements of eGFR were 4.37 years.

Positive values decline in eGFR, and negative values increase.

**Table 5:** Associations between TNFR1 and TNFR2 at baseline and eGFR difference (in % decrease per month) and risk of being a rapid eGFR-decliner (defined as quartile 4 of eGFR difference), respectively.

	eGFR difference		eGFR difference		
	Continuous	*P*	Quartiles 1–3	Quartile 4	*P*
	Regression coefficient (95% CI)		ref	OR (95% CI)	
*n*	455		342	113	
**TNFR1**					
Unadjusted model	0.0021 (0.0009–0.0033)	0.001	ref	1.71 (1.33–2.21)	<0.001
Model A (age, gender)	0.0023 (0.0011–0.0035)	<0.001	ref	1.76 (1.37–2.28)	<0.001
Model B (inflammation)	0.0023 (0.0011–0.0035)	<0.001	ref	1.74 (1.35–2.25)	<0.001
Model C (CVD risk factors)	0.0022 (0.0009–0.0033)	<0.001	ref	1.79 (1.38–2.33)	<0.001
Model D (A+B+C combined)	0.0021 (0.0009–0.0034)	0.002	ref	1.77 (1.35–2.30)	<0.001
Model E (Model D + baseline eGFR)	0.0012 (−0.0003 to 0.0027)	0.12	ref	1.29 (0.92–1.81)	0.14
**TNFR2**					
Unadjusted model	0.0023 (0.0011–0.0036)	<0.001	ref	1.87 (1.43–2.45)	<0.001
Model A (age, gender)	0.0026 (0.0014–0.0039)	<0.001	ref	1.97 (1.50–2.60)	<0.001
Model B (inflammation)	0.0027 (0.0014–0.0039)	<0.001	ref	1.95 (1.47–2.59)	<0.001
Model C (CVD risk factors)	0.0024 (0.0011–0.0037)	<0.001	ref	1.97 (1.49–2.62)	<0.001
Model D (A+B+C combined)	0.0024 (0.0011–0.0037)	<0.001	ref	1.94 (1.45–2.60)	<0.001
Model E (Model D + baseline eGFR)	0.0009 (−0.0009 to 0.0027)	0.322	ref	1.33 (0.90–1.98)	0.148

HR: hazard ratio; CI: confidence interval; CVD: cardiovascular disease; eGFR: estimated glomerular filtration rate.

eGFR based on cystatin C was also calculated using the CAPA equation. The differences in time between the measurements of eGFR were 4.37 years.

Model A: Adjusted for age and gender. Model B: Adjusted for age, gender, and C-reactive protein. Model C: Adjusted for age, gender, systolic blood pressure, diastolic blood pressure, smoking status, heart failure, diabetes, and prevalent cardiovascular disease.

Model D: Model A + B + C combined. Model E: Model A+B+C+baseline eGFR and u-protein.

## Discussion

The main findings of this study were that TNFR2 was associated with higher mortality, but that TNFR1 showed borderline significant associations, and that neither TNFR1 nor TNFR2 was associated with eGFR decline in patients with CKD when adjusting also for baseline eGFR and urine-protein. The results were overall similar for TNFR1 and TNFR2, which is not surprising as they are highly correlated variables.

Earlier studies have shown that both higher levels of TNFR1 (1, 2) and TNFR2 (3) are associated with higher mortality, but studies of mortality risk based on TNFR1 and TNFR2 in patients with CKD are sparce. We found TNFR2 to be associated with higher mortality, especially in the highest concentration quartile versus quartile 1, while TNFR1 only showed borderline significant values after adjusting also for baseline eGFR and U-protein.

Both TNFR1 and TNFR2 have been associated with decline in eGFR (3, 12), and the association between TNFRs and kidney function among the elderly has also been described previously (4, 5), but studies in patients or cohorts with CKD are as far as we know missing. It is possible that findings in population-based cohorts between TNFR1 and TNFR2 are attributed to kidney decline in patients with eGFR above 60, which makes our contribution novel.

The reason that we a priori defined relative eGFR decline as the primary outcome was because it normalizes the rate of decline relative to the individual’s baseline GFR. This is particularly useful when comparing eGFR decline in individuals with a wide variety of baseline kidney function as absolute changes in eGFR will have different clinical meaning in those with higher eGFR versus those with low eGFR. Also, relative decline in eGFR has been defined as a surrogate endpoint for end-stage kidney disease (ESKD) in clinical trials by both European Medicines Agency (EMA) and Food and Drug Administration (FDA) (definition >40% decline in eGFR). We found that the association between TNFR1 and TNFR2 and decline in eGFR disappeared when also adjusting for baseline eGFR and U-protein.

As TNF alpha is produced in macrophages/monocytes during acute inflammation and, thus, is involved in many signaling events within cells through TNFR, mainly leading to necrosis or apoptosis, the association with mortality and declining kidney function is likely to be mediated through inflammatory mechanisms (8). However, the results remained unchanged even after adjusting for CRP.

The association between TNFR and mortality has previously been described (1–3), in both cardiovascular and cancer mortality. Regarding cardiovascular diseases, inflammation is known to promote atherosclerosis (18), thus coronary artery disease, including mortality (19, 20). Furthermore, diabetes is a strong risk factor for cardiovascular complications (21), and TNFR is also linked to insulin resistance (22). In addition, TNFR and CKD also contribute to premature mortality in diabetes (23, 24).

The association between TNFR and CKD has been reported previously (3–5), also in individuals with diabetes (23–25), and higher TNFR contributes to the all-cause mortality (23, 24). However, in the present study, when also adjusting for baseline eGFR and U-protein for the mortality risk, TNFR1 was unsignificant, and for TNFR2, the risk estimate decreased but still on a significant level, thus showing the importance of baseline eGFR and U-protein.

There are some limitations with the study. The cohort included referred patients, many with advanced CKD, and we cannot generalize our findings to other age-, or ethnic groups or to other clinical settings. Most patients (97%) in the Salford Kidney Study are White. Only 3% are non-White patients. We did not have full access to the dates of cardiovascular and non-cardiovascular events and could not add separate analyses. Yet, the annual mortality rate is 9%, and the annual rate of individuals going into RRT in the Salford Kidney study is 5% (26), comparable to CKD prognosis consortium, which involved 28 cohorts totaling over 185,000 patients (27). The use of baseline eGFR while studying eGFR decline has been questioned (17). To use baseline eGFR when predicting the eGFR-decline has also been questioned previously by Carlsson et al (28). Furthermore, the rate of eGFR decline in patients with CKD varies according to age, underlying cause of CKD and degree of proteinuria. The annual eGFR decline rate was between 1 and 2 ml/min in the present study, similar to those demonstrated in an earlier individual patient meta-analysis of 1.7 million patients in the CKD Prognosis Consortium (29). Another limitation is the fact that hyperfiltration can occur prior to the development of diabetes (30). We were not able to assess the underlying cause of CKD and could not analyze immunosuppressants or corticosteroids that may be the cause of lupus-nephritis. It is possible that the results could have been influenced by a possible immune-mediated disease, for example, lupus nephritis, but we judge that this could have affected the result only marginally, as this is an unusual cause of CKD. Albuminuria is clinically used, whereas we used U-protein. However, U-protein over 24 h is often regarded as a gold standard and is often used in trials.

In conclusion, in this secondary care cohort of individuals with CKD, higher plasma levels of TNFR2 were associated with an increased risk of mortality, while the association between plasma TNFR1 and mortality did not pass the significance threshold. There was no association between the TNFRs with further decline in kidney function when adjusting for baseline eGFR and U-protein. TNFR1 and TNFR2 portray interesting aspects in patients with CKD, but the clinical utility seems limited.

## Supplementary Material


