# Supplementary material for: The association between TNF-receptors (TNFR1 and TNFR2) and mortality as well as kidney function decline in patients with chronic kidney disease
Source: Ups J Med Sci. 2024 Nov 25;129:10.48101/ujms.v129.10726. doi: 10.48101/ujms.v129.10726 (PMC11653433; doi:10.48101/ujms.v129.10726)
Supplement: Supplementary file 1 [file UJMS-129-10726-s1.pdf]

Supplementary files.

Supplementary Figure 1. **Nelson-Aalen plot of cumulative incidence of mortality by participants divided in quartiles according to concentrations of TNFR1.**

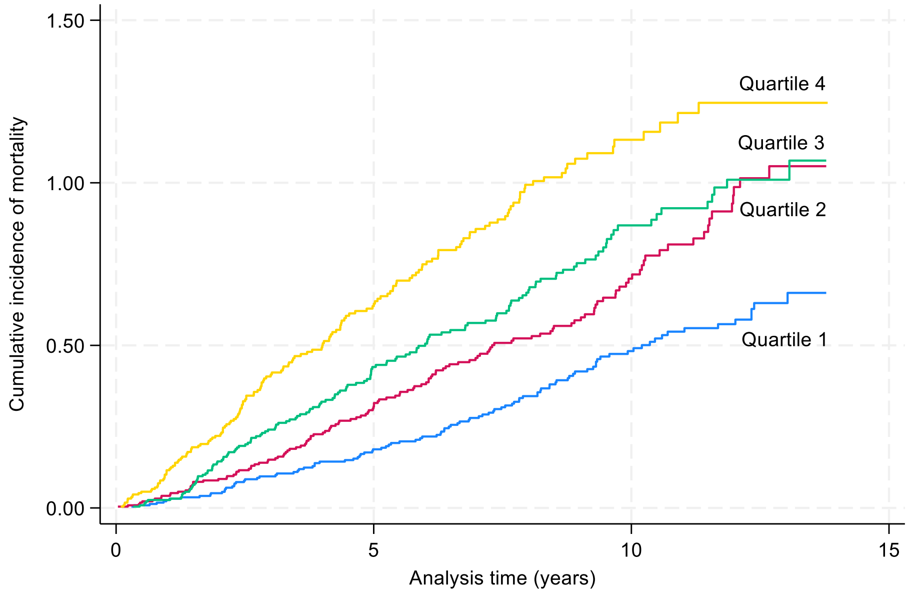

Number at risk				
Quartile 1	248	207	114	0
Quartile 2	246	180	77	0
Quartile 3	247	159	61	0
Quartile 4	246	132	45	0

Supplementary Figure 2. **Nelson-Aalen plot of cumulative incidence of mortality by participants divided in quartiles according to concentrations of TNFR2.**

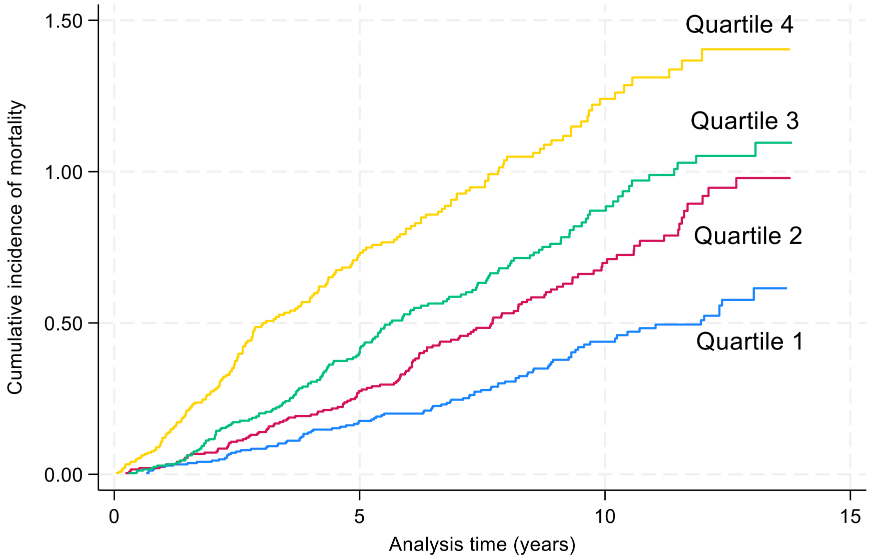

Number at risk				
Quartile 1	247	207	99	0
Quartile 2	245	186	79	0
Quartile 3	246	163	69	0
Quartile 4	246	119	50	0
